# Preparation of Chitosan/Polyacrylamide/Graphene Oxide Composite Membranes and Study of Their Methylene Blue Adsorption Properties

**DOI:** 10.3390/ma13194407

**Published:** 2020-10-02

**Authors:** Zheqi Wang, Guohua Zhang, Yanhui Li

**Affiliations:** 1 College of Mechanical and Electrical Engineering, Qingdao University, Qingdao 266071, China; wangzheqiqdu@outlook.com; 2 State Key Laboratory of Bio-Fibers and Eco-Textiles, Collaborative Innovation Center for Marine Biomass Fibers Materials and Textiles of Shandong Province, Qingdao University, Qingdao 266071, China

**Keywords:** graphene oxide, chitosan, synthetic material, adsorption, methylene blue

## Abstract

This thesis reports the preparation of chitosan/polyacrylamide/graphene oxide nanocomposites (CAGs) and a study of its adsorption properties of methylene blue (MB) solution. Initially, we synthesized CAGs by blending and freeze-drying methods. Then, we conducted a series experiments by removing MB from aqueous solution to test its adsorption properties and adsorption mechanism. We used UV-Vis spectrophotometry to determine the concentration of residual methylene blue accurately and efficiently, which has a specific absorption peak at 662 nm in the UV-Vis spectrum, in aqueous solution. When the graphene oxide content in the composite was 20 wt%, the adsorption capacity reached maximum values. The chemical properties and surface structure of the nanomaterials were analyzed using FT-IR, TGA, SEM, and BET. Also, we carried out experiments to measure the adsorption properties of the CAGs by varying several factors, such as initial concentration, adsorption time, and pH, etc. The outcomes revealed that the adsorption equilibrium was developed after 2800 min at 20 °C (room temperature) with an adsorbent dosage of 0.01 g mL^−1^. The ion adsorption equilibrium data were well-fitted by the Langmuir isotherm with a maximum monolayer capacity of 510.2 mg/g. Kinetic researches disclosed that the adsorption procedure was defined by a pseudo-second-order model. Thermodynamic researches revealed that the enthalpy change (Δ*H* ^0^) as well as Gibbs free energy change (Δ*G* ^0^) of the adsorption procedure was negative, indicating that the adsorption procedure was spontaneous and exothermic. After three cycles, the removal efficiency was still 90.18%. Therefore, in conclusion, we believe that the CAGs is a good adsorption material for organic dyes due to its good adsorption and recyclable properties.

## 1. Introduction

In recent years, along with progress of society and the development of technology, the change of the dye industry is huge. On the one hand, some researchers have worked on synthesizing dyes through optoelectronic means [1], thus significantly reducing the difficulty of dye manufacturing. On the other hand, besides the demand for dyes becoming greater due to advances in the textile, dyeing, and printing industries [2], many dyestuffs play a new role nowadays. For example, methylene blue (MB), a traditional dye, has been commonly used in medical treatments in recent years [3]. However, the extensive use of dyes is generating a large amount of dye effluent, which has a tremendous impact on water resources and the ecological environment. Moreover, long-term ingestion of water containing dyes can cause trauma to the human liver, the digestive and central nervous systems, and can even lead to cancer [4]. As mentioned earlier, methylene blue is a typical and commonly used industrial dye, however its biological toxicity, caused by its stable and complex benzene ring structure and non-degradability [5], limits its further applications. As a kind of nanomaterial, graphene has a larger specific surface area than similar materials. At the same time, due to its strong chemical stability and excellent electrical and thermal properties, it has been widely used in various fields [6], e.g., as a metal catalyst [7]. However, graphene also has disadvantages. In the sheet form, its surface area and adsorption capacity are greatly reduced due to its insolubility and tendency to aggregate in aqueous environments [8]. However, graphene oxide (GO) is quite different. The GO contains a large amount of oxygen-containing functional groups, i.e., epoxy, hydroxyl as well as carbonyl [9]. As a result, it will work well in many fields, such as DNA detection [10], cathodic protection [11,12], graphene transistors [13], and environmental protection, that can absorb the heavy metal ions and organic pollutants from polluted wastewater [14,15]. Lots of researchers have produced different GO-based materials as adsorbents for removing the heavy metal ions and organic dyes in the last few years. For example, researchers have employed a kind of porous GO material to remove Pb (II) from wastewater [16], a new chitosan/GO polymer as an adsorbent for the adsorption of arsenic [17], and magnesium ferrites/GO to adsorb Ni (II) and Pb (II) ions [18]. It is still unclear whether GO is a suitable adsorption material with high adsorption affinity. However, GO disperses well in water and requires a long time to separate from solution after adsorption, which limits its practical use. To solve this problem, we have combined GO with other polymers or inorganic composites to form a composite material that can stabilize GO in water. This composite material not only contains the excellent properties of GO, but is also not easily dispersed in solution, which is convenient for recycling. In this way, the adsorption capacity of GO has greatly enhanced. Meanwhile, it can also play a good role in environmental protection.

## 2. Materials and Methods

### 2.1. Chemical and Reagents

The expanded graphite (300-mesh) used in the experiment was purchased from Qingdao Henglide Graphite Co., Ltd. (Qingdao, China). In addition, acrylamide (AM) (CH_2_=CHCONH_2_), chitosan (CS), potassium permanganate (KMnO_4_), hydrogen peroxide (H_2_O_2_), sodium nitrate (NaNO_3_), 30 wt% diluted hydrochloric acid (HCl), concentrated sulfuric acid 98 wt% (H_2_SO_4_), *N*,*N*,*N*′,*N*′-tetramethylethylenediamine (TEMED) ((CH_3_)_2_NCH_2_CH_2_N(CH_3_)_2_), and potassium persulfate (KPS) were all purchased from Aladdin Reagent (Qingdao, China).

### 2.2. Preparation of CAG

This experiment used an improved Hummers method to prepare GO [19].

The synthesis method of the chitosan/polyacrylamide/graphene oxide nanocomposite (CAG) was as follows. First, we weighed different amounts of GO and added them into the aqueous solution respectively as Table 1 shows. Second, as shown in Table 1, we added different amounts of chitosan, acrylamide to the GO aqueous solution respectively. The CAG solutions were got after 12 h constant stirring. Last, we maintained the temperature of the CAG solutions at 0 °C and added a certain amount of KPS and TEMED sequentially. After stirring for 1 h at 0 °C, the mixture was maintained at room temperature for 24 h to obtain a CAG oxide hydrogel, which was freeze-dried for 12 h to obtain the dry composite material [20].

### 2.3. Method for Experiment

#### 2.3.1. Method for Measuring the Methylene Blue Concentration

A certain amount of MB was weighed. Then, we prepared an MB solution with a concentration of 1000 mg/L, which was diluted to 1 mg/L, 3 mg/L, 5 mg/L, 7 mg/L, and 9 mg/L respectively. The standard curve of MB was obtained by measuring the transmittance of these solutions and linear fitting. The standard curve was:

(1) Conc=9.98Abs−0.05

where *Conc* (mg/L) represents the concentration of MB, *Abs* represents absorbance.

Because when the absorbance is greater than 1, the measurement result will become inaccurate, it is necessary to dilute the adsorbed solution 10 times to get accurate values. Since dilution would cause errors in general, we diluted it 3 times and took the average value of the solution to eliminate the error.

After the dye solutions of different concentrations were adsorbed by the composite material, the residual MB solution was diluted and then measured by an ultraviolet spectrophotometer (Thermo Fisher, Waltham, MA, USA). The formulas for calculating the adsorption capacity and removal rate *r*% at time *t* (min) are as follows:

(2) $$q_{e}=\frac{\left(c_{0}-c_{e}\right)\times V}{W}$$

(3) $$q_{t}=\frac{\left(c_{0}-c_{t}\right)\times V}{W}$$

(4) $$r\%=\left(\frac{c_{0}-c_{e}}{c_{0}}\right)\times 100\%$$

where *c*_0_ (unit: mg/L) represents the initial concentration of MB, *c_e_* (unit: mg/L) represents the concentration at the reaction equilibrium, *c_t_* (unit: mg/L) represents the concentration at time *t* (min), *V* (unit: mL) represents the volume of the solution, and *W* (unit: mg) represents the adsorbent weight.

#### 2.3.2. Influence of GO Amounts of CAG

In this experiment, we added CAG materials (10 mg) with different GO proportions to 20 mL of MB solution at a concentration of 100 mg/L and measured the concentration of the remaining MB solution at reaction equilibrium, which was reached after constant shaking at room temperature (20 °C) for 48 h.

#### 2.3.3. Influence of Different Quantities of CAG

In this experiment, we added different amounts of CAG, that were 5 mg, 10 mg, 15 mg, 20 mg, and 25 mg, to 20 mL of MB solution at a concentration of 200 mg/L respectively. Then, the concentration of the remaining MB solution was measured after constant shaking at room temperature (20 °C) for 48 h.

#### 2.3.4. Influence of the pH Value of MB Solution

In this experiment, we first prepared 10 bottles MB solution, that containing 20 mL with the concentration of 200 mg/L respectively. Then, we added nitric acid (HNO_3_) or sodium hydroxide (NaOH) to the abovementioned MB solution to obtain MB solutions with different pH values of 1, 2, 3, 4, 5, 6, 7, 8, 9, and 10. Then, we added 10 mg of CAG to an MB solution with a given pH value and measured the concentration of the remaining MB solution after constant shaking at 20 °C for 48 h.

#### 2.3.5. Influence of the Temperature and Concentration of MB Solution

In this experiment, we added 10 mg of CAG to MB solutions with different MB concentrations, of which 120 mg/L, 140 mg/L, 160 mg/L, 180 mg/L, 200 mg/L, 220 mg/L, 240 mg/L, 260 mg/L, 280 mg/L, 300 mg/L, and 320 mg/L. Then, these 11 solutions were maintained at different constant temperatures of 293 K, 313 K and 333 K for 48 h. After reaching adsorption equilibrium, we measured the concentration of the remaining MB solution.

#### 2.3.6. Influence of Adsorption Time

In this experiment, we added 150 mg of CAG into 300 mL of a 300 mg/L MB solution and measured the MB concentration in the solution at given time intervals until the reaction equilibrated after 48 h.

### 2.4. Characterization Method

The characterization of CAGs was carried out by FT-IR spectra, scanning electron microscopy (SEM), thermogravimetic analysis (TGA), and then tested for BET. For the FT-IR spectra, we measured it by a Nicolet 5700 spectrometer (Thermo fisher, Waltham, MA, USA) at room temperature. For the SEM, we have got a cross-sectional image through a Quanta FEG 250 instrument (FEI Company, Hillsboro, OR, USA) at a voltage of 10 kV. X-ray diffraction (XRD) patterns were recorded with a DX-2700 (Shanghai Precision Scientific Instruments Co., Shanghai, China) from 5° to 80° with intervals of 0.02° $s^{-1}$. For the TGA, we conducted it by an SDT650 thermogravimetric analyzer (TA Discovery, New Castle, DE, USA). The sample CAG was heated from 30 °C to 800 °C, that at a heating rate of 10 °C min^−1^ and maintained at 800 °C for 1 min. Finally, for the BET of CAG, we analyzed the specific surface area of the CAG through an ASAP 2460 specific surface area analyzer (American Micromeritics Company, New Castle, DE, USA).

## 3. Characterization of CAG Nanoparticles

**Figure 1:**
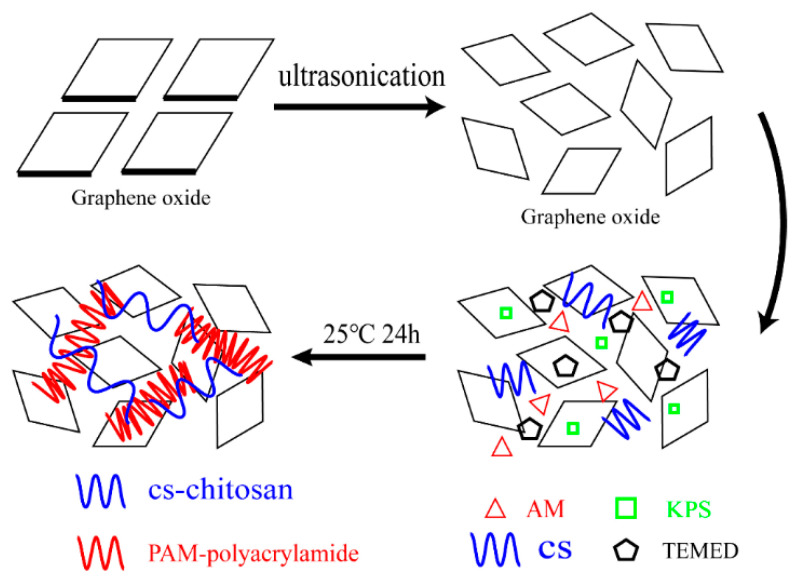
Synthetic process of CAG nanoparticles.

As shown in Figure 1, this experiment synthesized a chitosan/polyacrylamide/graphene oxide nanocomposite (CAG) using the blending method. To ensure the uniform dispersion of GO, it was mixed in water and ultrasonicated for 30 min to disperse the polymerized GO. According to the XRD curve, the characteristic peak at 2θ = 10.9°, which was the GO nanosheets located, and the corresponding interlayer distance was at 0.810 nm, which also shows that most GO nanosheets formed monolayers [21]. In the present work we decided to prepare a family of CAG nanoparticles by keeping fixed the quantity of chitosan and polyacrylamide, CS and AM were fixed at 300 mg and 1 g, respectively. And varying the load of GO: 0 wt%, 5 wt%, 10 wt%, 15 wt%, 20 wt%, and 25 wt%. During the experiment, KPS acted as an initiator and TEMED acted as a crosslinker. It was found that when the GO content exceeded 20 wt%, the freeze-dried material appeared powdery and the composite material did not form, which could be because there was too much GO to completely react with the other materials.

### 3.1. SEM Analysis

**Figure 2:**
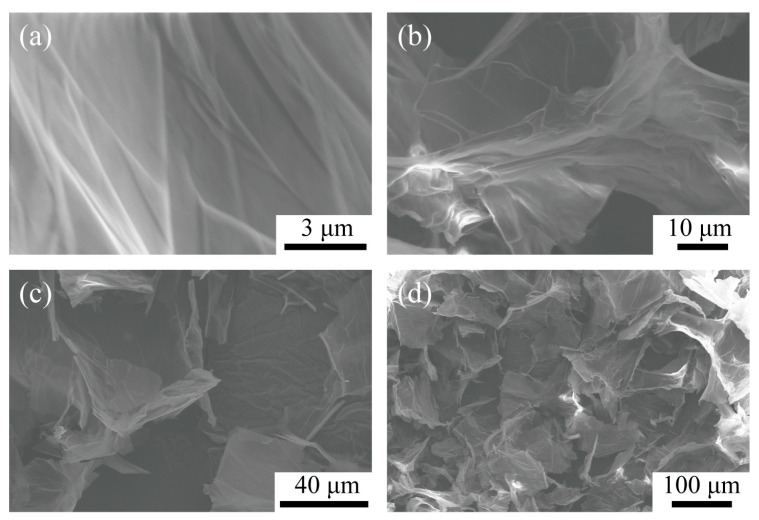
SEM images of CAG-GO20%. (**a**) Magnify 30,000 times. (**b**) Magnify 7000 times. (**c**) Magnify 2500 times. (**d**) Magnify 680 times.

Figure 2 shows a SEM image of the synthesized CAG material. It can be clearly seen that the material has a large number of folds, which greatly increases the specific surface area of the material, and accordingly provide lots of adsorption sites for the adsorption of MB [22].

### 3.2. XRD Analysis

**Figure 3:**
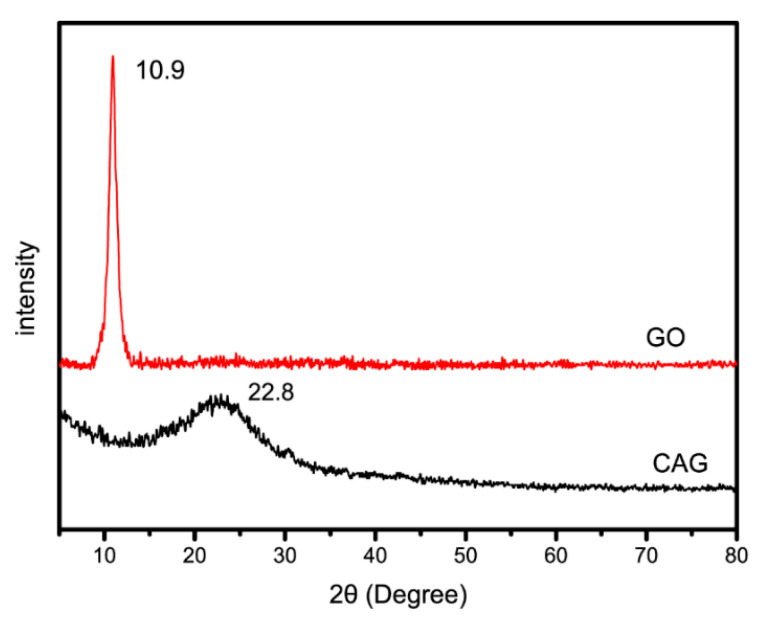
XRD spectra of CAG-GO20%.

As shown in Figure 3, the abscissa is the peak position, that is, the 2θ position of the diffraction peak. The ordinate is the peak height, representing the peak height intensity, in the XRD pattern of the GO nanosheets, a narrow strong diffraction peak was detected at 2θ = 10.9°, corresponding to an interlayer distance of 0.810 nm, which was consistent with the reported literature [23]. However, in the synthetic composite material CAG-GO20%, a wider diffraction peak was detected at 2θ = 22.8°, while the strong diffraction peak of GO completely disappeared, indicating that the materials were successfully combined. This can also indicate that GO nanosheets are uniformly dispersed in the CAG material without coacervation, the material is amorphous, and GO lost its original structure.

### 3.3. FT-IR Analysis

**Figure 4:**
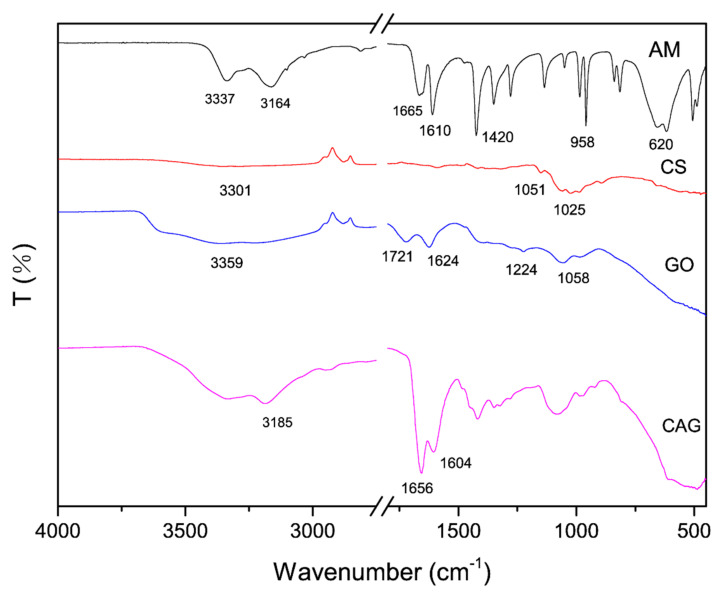
FT-IR spectra of AM, CS, GO, and CAG-GO20%.

Figure 4 shows FT-IR images of AM, CS, GO, and CAG-GO20%. Acrylamide peaks at 3337 cm^−1^, 3164 cm^−1^, 1665 cm^−1^, 1610 cm^−1^ and 1420 cm^−1^, 958 cm^−1^, and 620 cm^−1^ corresponding to the N-H stretching vibration, C=O stretching vibration, N-H in-plane bending vibration, C-N stretching vibration, and NH_2_ in-plane and out-plane rocking, respectively, were observed in the infrared spectrum of AM. 

The broad peak at 3400–3200 cm^−1^ of chitosan (CS) involves O-H and N-H stretching vibrations, and the characteristic peak of sugar structure at 1051^−1^ and 1025 cm^−1^. The peaks at 3359 cm^−1^, 1721 cm^−1^, 1624 cm^−1^, 1224 cm^−1^, and 1224 cm^−1^ of GO were attributed to the stretching vibration of -OH, the stretching vibration of C=O of the -COOH group, the C=C stretching vibration of the sp^2^-hybrid carbon chain, and the C-OH stretching vibration of the -COOH group, respectively [24]. The absorption peak at 1058 cm^−1^ was related to the C-O-C flexion and extension vibration stretching vibration [25]. 

Comparing the FT-IR spectra of AM and the synthesized CAG material, the diffraction peak of N-H stretching vibration originally located at 3164 cm^−1^ moved to 3185 cm^−1^. The diffraction peak related to C=O stretching vibration of amide group moved to CAG at 1665 cm^−1^. The characteristic peak of in-plane bending vibration at 1610 cm^−1^ of N-H moved to 1604 cm^−1^ in CAG. Comparing GO and the synthesized CAG material, the characteristic peak corresponding to O-H at 3185 cm^−1^ is significantly broadened by the addition of GO; and the characteristic peak corresponding to C=O stretching vibration at 1721 cm^−1^ is also moved to 1656 cm^−1^ in the CAG material. At this point, the shift of the absorption peak of the amide group and the wider peak of the O-H group indicate the existence of a large number of hydrogen bonds in the composition. The CAG spectrum contained all the characteristic peaks of AM, CS, and GO. These results show that the materials were successfully combined and exhibited the corresponding functional groups.

### 3.4. BET Analysis

**Figure 5:**
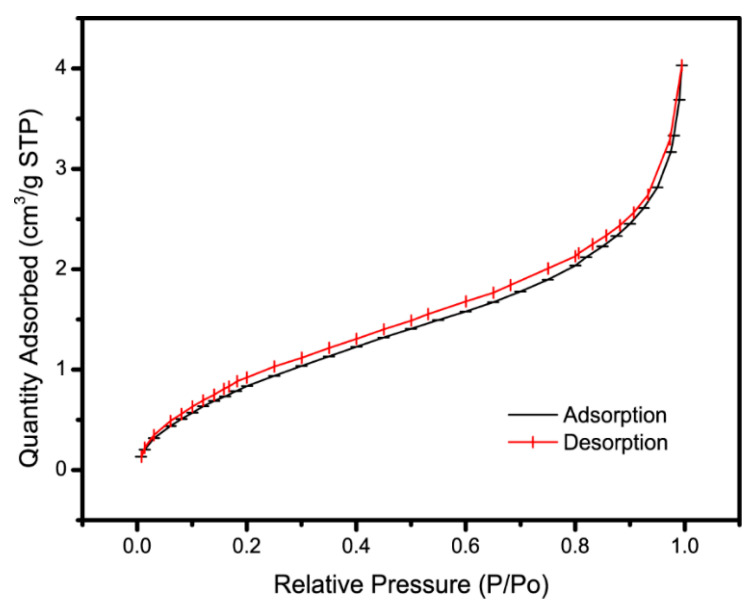
BET of CAG-GO20%.

As shown in Figure 5, after the BET test of the CAG-GO20% nanocomposite, its specific surface area was found to be 3.7603 m^2^/g, it belongs to type II isotherm. Single point surface area at P/Po = 0.250057140, which is the first steep part of the isotherm, it indicates the saturated adsorption capacity of the monolayer, which is equivalent to the completion of the monolayer adsorption. With the increase of relative pressure, a long and slowly rising platform appeared, which indicated that the adsorption gradually diffused from the surface monolayer to the inner molecular layer, and multilayer adsorption began. When the relative pressure is high, capillary condensation occurs.

### 3.5. TGA Analysis

**Figure 6:**
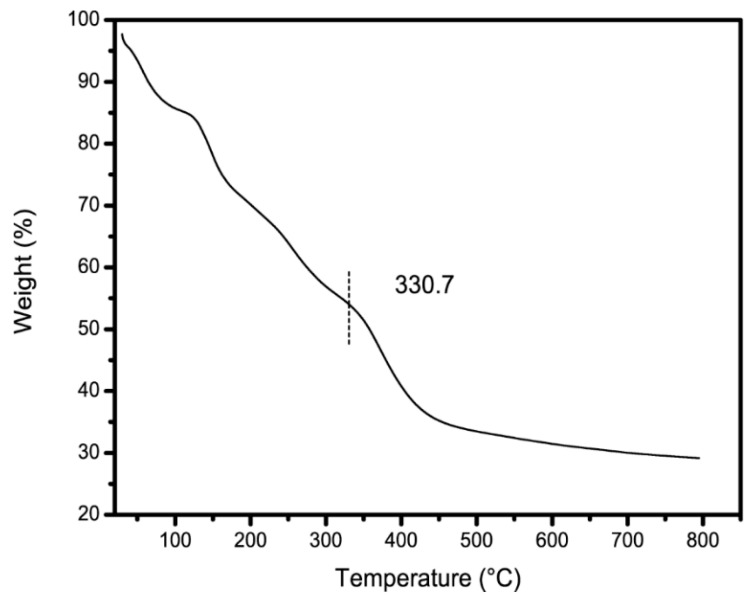
TGA of CAG-GO20%.

As shown in Figure 6, when CAG-GO20% was heated from 30 °C to 800 °C, the curve initially reduced relatively smoothly. This decrease was due to the gradual loss of water in the material. When the temperature increased to 330.7 °C, the rate of weight loss increased significantly. The main reason for this reduction was the decomposition of certain functional groups, such as hydroxyl and epoxy groups, in the high-temperature environment. As the temperature increased further, after 420 °C, the rate of weight loss decreases, and carbons in the carbon chains were gradually decomposed until a stable level was reached [26]. When the temperature reached 800 °C, the residual amount of the material was 29.16%.

## 4. Methylene Blue Adsorption Properties

In this part, we varied the proportion of GO of CAG, quantity of CAG, temperature and concentration of the MB solution and conducted a series of experiments. At the same time, we fitted the experimental data to the different adsorption models and conducted research on the adsorption kinetics, adsorption thermodynamics, and adsorption isotherms to illustrate the relevant properties of CAG.

### 4.1. Influence of Different GO Proportions of CAG

**Figure 7:**
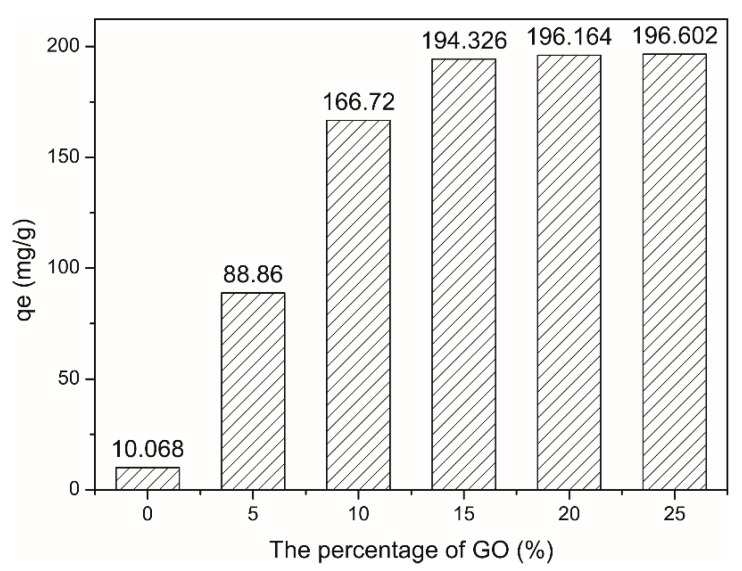
Percentage of GO, MB solution: 20 mL, 100 mg/L, Room temperature (20 °C).

Figure 7 shows the effect of different GO proportions of CAG on the adsorption performance. The *x*-axis represents the percentage of GO in the total mass of the material, and the *y*-axis represents the adsorption capacity. When the GO proportion was 0 wt%, the adsorption capacity was only 10.068 mg/g, indicating a poor adsorption performance. As the GO proportion in CAG increased, the adsorption performance improved. When the GO proportion reached 15 wt%, the adsorption capacity was 194.326 mg/g, indicating that an increase in the GO proportion greatly increased the CAG adsorption performance. When the GO proportion exceeded 15 wt%, the adsorption capacity did not increase significantly. This is because the maximum adsorption capacity had already been reached. Therefore, the higher the GO proportion in the CAG, the better the adsorption performance. However, when the GO proportion reached 25 wt%, CAG generated a large amount of powder, which is not a desired feature for solution adsorption. Therefore, we used CAG with 20 wt% GO in subsequent experiments.

### 4.2. Influence of Different Quantities of CAG

**Figure 8:**
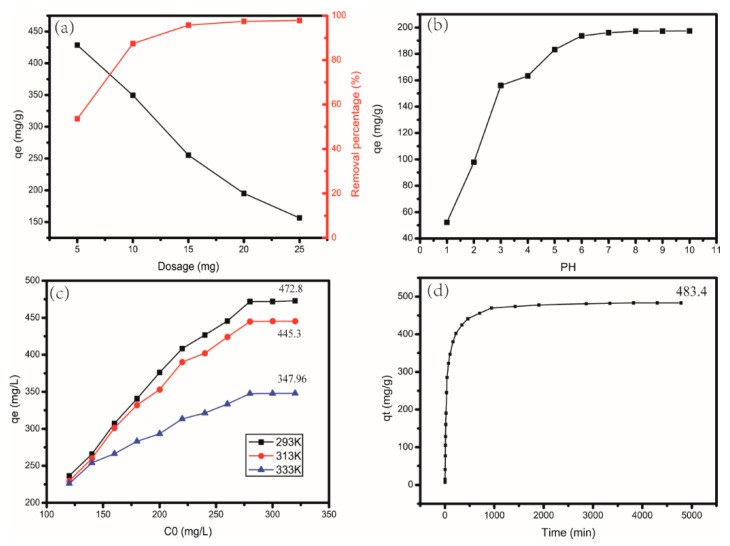
Effect of (**a**) dosage at room temperature (20 °C), MB solution: 20 mL, 200 mg/L, (**b**) pH 1–10, MB solution: 20 mL, 100 mg/L, room temperature (20 °C), (**c**) temperature, MB solution: 20 mL, 120–320 mg/L, (**d**) time, amount of CAG: 150 mg, MB solution: 300 mL, 300 mg/L, room temperature (20 °C) on adsorption.

As shown in Figure 8a, the *x*-axis represents the initial amounts of CAG, the left-y axis represents the adsorption capacity, and the right y-axis represents the removal percentage, the black line represents the relationship between the adsorption capacity and the amount of CAG material added, and the red line represents the relationship between the removal percentage and the amount of CAG material added.

The adsorption process was greatly influenced by the amount of CAG. With the increase in CAG, the removal rate also increased. This behavior was because, due to an increase in the amount of CAG, adsorption sites also increased significantly. However, the adsorption capacity decreased with an increasing amount of adsorbent. Because the number of adsorption sites increased, their utilization rates decreased at the same time, which resulted in a decrease in the adsorption capacity.

### 4.3. Influence of the pH Value of MB Solution

The results shown in Figure 8b, the *x*-axis represents the initial pH of the solution, and the *y*-axis represents the adsorption capacity, that when the pH increased from 1 to 4, the adsorption capacity increased significantly, and the removal rate also increased. This behavior can be attributed to the fact that at low pH values, H^+^ in the solution competes with methylene blue for adsorption sites, which causes a decrease in the adsorption efficiency. Another reason for this behavior is that the molecular electrostatic attraction forces between the composite membrane and MB are reduced at low pH [27]. As shown in Figure 8b, increasing the pH further had little effect on the adsorption effect.

### 4.4. Influence of the Temperature and Concentration of MB Solution

As shown in Figure 8c, the *x*-axis represents the concentration of the initial MB solution, and the *y*-axis represents the adsorption capacity. Different colored lines represent different ambient temperatures, at an initial concentration of less than 160 mg/L, the temperature did not affect the adsorption capacity, but after a concentration higher than 160 mg/L, the adsorption capacity changed significantly at different temperatures, at a temperature of 293 K, the adsorption capacity also increased with increasing MB solution concentration. As the initial concentration of the solution increased from 120 mg/L to 320 mg/L, the adsorption capacity also increased from 236.5 mg/g to 472.8 mg/g. At the same concentration of 340 mg/L, the adsorption capacity rapidly decreased from 472.8 mg/g to 347.96 mg/g when the temperature increased from 293 K to 333 K, indicating that the adsorption process of CAG with MB was an exothermic process.

### 4.5. Influence of Adsorption Time

As shown in Figure 8d, the *x*-axis represents time, and the *y*-axis represents the adsorption capacity at time t, in the initial stage of adsorption, the adsorption capacity increased rapidly. With increasing time, the increase in adsorption capacity decreased until it reached equilibrium.

This is because, during the initial stage, there are a large number of active adsorption sites on the material. With ongoing adsorption, these adsorption sites are gradually saturated, and the increase in the adsorption capacity of CAG gradually becomes slower. The diffusion of MB from the surface into the interior also requires some time. This diffusion is another reason for why it took more than 48 h to reach adsorption equilibrium.

## 5. Adsorption Model Analysis

### 5.1. Adsorption Isotherm

The adsorption isotherm model is used to analyze the reaction mechanism between an adsorbent and an adsorbate, The Langmuir and Freundlich models are the two most commonly used models. The linear formula for the Langmuir model is [28] as follows:

(5) $$\frac{C_{e}}{q_{e}}=\frac{C_{e}}{q_{max}}+\frac{1}{q_{max}K_{L}}$$

**Figure 9:**
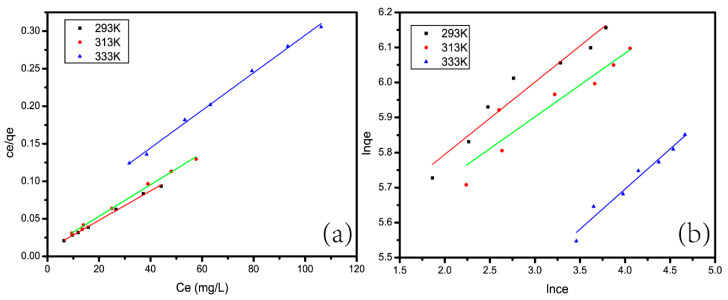
Langmuir plot (**a**) and (**b**) Freundlich plot.

In the above formular, *K_L_* (L/mg) represents the Langmuir constant and *q_max_* (mg/g) is the maximum adsorption capacity. Through comparison and calculation using the Langmuir model, we obtained the curve shown in Figure 9a. In Figure 9a, the *x*-axis represents *C_e_* and the y-axis represents *C_e_*/*q_e_*. We got the specific parameters by linear fitting the detailed values of *q_max_* and *K_L_*. The results were listed in Table 2 As we can see, for the Langmuir model, the maximum theoretical adsorption capacities of CAG at 293 K, 313 K and 333 K were 510.2 mg/g, 476.19 mg/g, and 400.28 mg/g, respectively, which were similar to the experimental data. The correlation coefficients R^2^ were all greater than 0.99. It can be concluded that the experimental data fit the Langmuir model well.

We have also investigated the adsorption effect of synthetic materials containing graphene oxide, polyacrylamide, or chitosan on methylene blue. As listed in the Table 3, because the maximum theoretical adsorption capacity of CAG is 510.2 mg/g, the effect of CAG on MB adsorption is better than most other materials, but lower than the Chitosan/Graphene Oxide Nanocomposites. This shows that CAG is a good material for MB removal, but there is still room for improvement.

Another formula for the Langmuir model is:

(6) $$R_{L}=\frac{1}{1+C_{0}k_{L}}$$

In the above formular, *k_L_* represents the Langmuir constant (L/mg) and *C*_0_ stands for the initial MB concentration (mg/L). In general, adsorption does not occur when *R_L_* > 1 and is favorable when 0 < *R_L_* < 1. Additionally, adsorption follows a linear trend when *R_L_* = 1 and is irreversible when *R_L_* = 0. Comparing the experimental results of this study, all the *R_L_* values obtained from the experiments were between 0 and 1, indicating that CAG had a high adsorption capacity for MB.

The formula for the Freundlich isotherm is [39]:

(7) $$lnq_{e}=lnk_{F}+\frac{1}{n}lnc_{e}$$

From Figure 9b, it can be seen that although the specific parameters can be linearly fitted, as shown in Table 2, the correlation coefficients *R*^2^ at 293 K, 313 K, and 333 K were 0.9337, 0.8523, and 0.9613, respectively, which were all less than 0.99. Through adsorption isotherm analysis, it can be concluded that the adsorption behavior of CAG can be described by the Langmuir model.

### 5.2. Adsorption Kinetics

We used several different kinetic models, i.e., pseudo-first-order, pseudo-second-order, and intraparticle diffusion models to describe the adsorption process and efficiency, for analyzing the MB adsorption. The results can be referred to Table 4.

The adsorption model of pseudo-first-order adsorption is [40]:

(8) $$\log\left(q_{e}-q_{t}\right)=logq_{e}-\frac{k_{1}}{2.303}t$$

of which the *q_e_* and *q_t_* stand for the adsorption capacity (mg/g) when the adsorption is completed at time *t* (min). The specific values of *k*_1_ and *q_e_* can be calculated.

**Figure 10:**
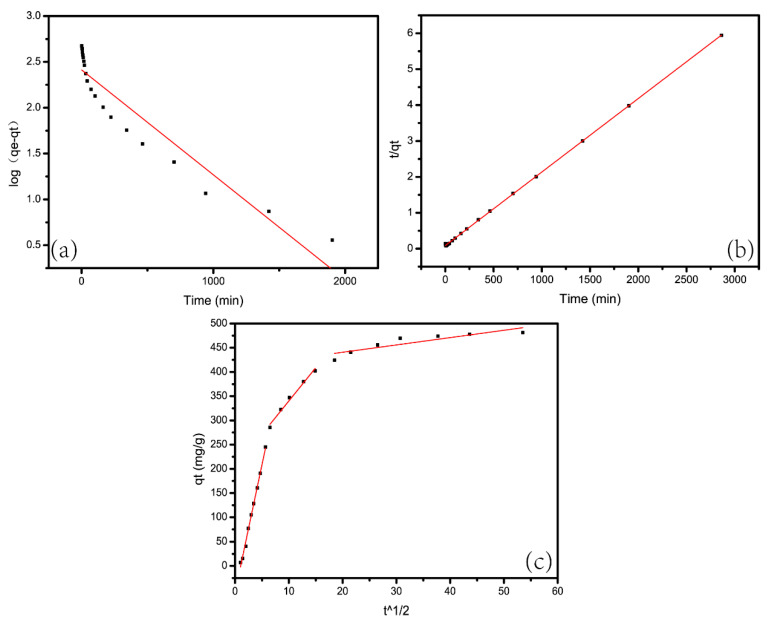
(**a**) Pseudo-first-order model, (**b**) pseudo-second-order model, (**c**) intraparticle diffusion model.

Fitted line of log(*q_e_* − *q_t_*) as a function of t (Figure 10a), as shown in Table 3. The correlation coefficient R^2^ obtained by fitting the data was only 0.8868. Therefore, the adsorption could not be described with the pseudo-first-order model.

The adsorption model of pseudo-second-order adsorption is [41]:

(9) $$\frac{t}{q_{t}}=\frac{1}{k_{2}q_{e}^{2}}+\frac{t}{q_{e}}$$

We calculated the values of *k*_2_ and *q_e_* through the intercept and slope of the linear fit of *t*/*q_t_* to *t*, which were shown in the Figure 10b respectively. Through calculations and comparisons, we find that the correlation coefficient of quasi-second-order kinetic equation model (*R*^2^), which equals to 0.9998, is greater than that of the quasi-first-order kinetic equation model which equals to 0.8868. Therefore, as conclusion, the dynamic adsorption data of CAG for MB conformed to the pseudo-second-order model.

The diffusion mechanism of the adsorption process is defined by the intraparticle diffusion model. Its mathematical expression is [42] as follows:

(10) $$q_{t}=k_{id}t^{1/2}+C$$

We calculated the intercept and slope of the linear fit of *t*^1/2^ as a function of *q_t_* and obtained the values of *k_id_* and *C*. Figure 10c shows that the fitted curve can be divided into three linear segments. Additionally, the intercept was not zero. In addition, these lines didn’t pass through the origin. Therefore, intraparticle diffusion was not the only dominant factor of adsorption. The boundary effects may also affect the adsorption kinetics of MB [43] in addition to intraparticle diffusion.

### 5.3. Adsorption Thermodynamics

Temperature is also an important factor which affecting adsorption. We acquired the enthalpy change (Δ*H* ^0^) and entropy change (Δ*S* ^0^) using the Van’t Hoff model [44]. At the same time, we measured the thermodynamic data of adsorbed MB at different temperatures, i.e., thermodynamic parameters. The formula of the model is as follows:

(11) $$\ln\left(\frac{q_{e}}{c_{e}}\right)=-\frac{\Delta H}{RT}+\frac{\Delta S}{R}$$

The formula we used to calculated the Gibbs free energy (Δ*G*) is as follows:

(12) ΔG=ΔH−TΔS

For the above formulas, gas constant (8.314 J/mol K) is represented by *R*, the absolute temperature (K) is represented by *T*. We performed a linear fit using the relevant thermodynamic data. –Δ*H* ^0^/*T* and Δ*S* ^0^/*R* were the parameters of the fitted graph. We have listed the specific thermodynamic data in Table 5.

As we can see, the adsorption process of CAG materials was a spontaneous reaction occurring without any external force [45] due to the all the value of Δ*G* ^0^ was negative. The values of Gibbs free energy (Δ*G* ^0^) increased from −5.94 kJ/mol to −2.23 kJ/mol, with the temperature increased from 293 K to 333 K, which revealing that the lower temperature favors the adsorption. Furthermore, the adsorption is an exothermic process due to the value of Δ*H* ^0^ was negative. 

## 6. Regeneration Research

**Figure 11:**
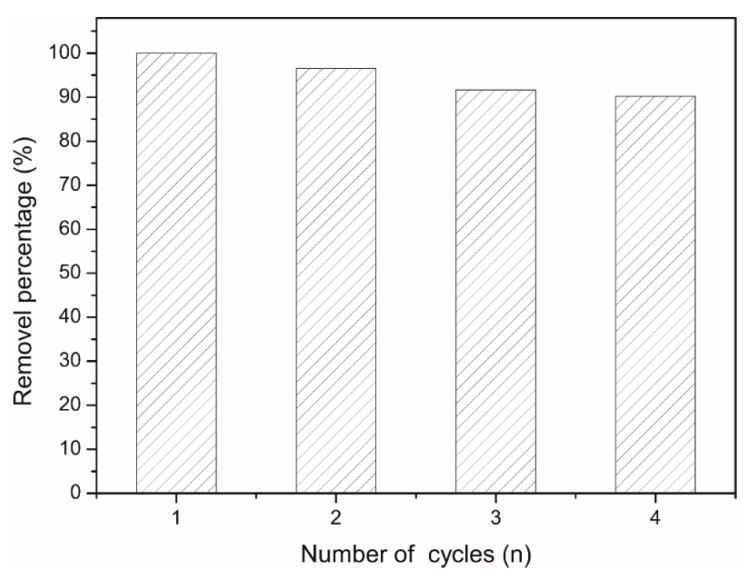
Number of cycles.

In this experiment, we first added 10 mg of CAG to 20 mL, 100 mg/L MB solution. After constant shaking at room temperature (20 °C) for 48 h, we measured the concentration of the remaining MB solution. Second, we removed CAG from the MB solution and used the excess 3 M HCl solution for desorption. Specifically, we added CAG to the excess 3 M HCl first and let it desorb under constant shaking at room temperature (20 °C) for 5 h. Next, for removing the excess HCl and MB, we washed CAGs with distilled water several times. Then, we added the CAGs, that after washing and freeze-drying for 24 h, to 20 mL 100 mg/L MB solution again for 48 h with constant shaking at room temperature. The above experimental steps were repeated several times, and the experimental results are displayed in Figure 11, where the *x*-axis stands for the number of cycles and the y-axis stands for the removal percentage.

After CAG underwent three adsorption-desorption processes, the MB solution removal rate was still 90.18%. Therefore, it can be concluded that CAG is an excellent recyclable dye adsorbent.

## 7. Research Results

(1) In the experiment, we prepared the graphene oxide (GO) through an improved Hummers method, which contained a large number of folded structures. Then, we prepared chitosan/polyacrylamide/graphene oxide nanocomposites (CAGs) by co-blending and freeze-drying. The SEM results indicated that the surface of CAG also contained many folded structures and was porous.

(2) Both the XRD and FT-IR results showed that the three raw materials, chitosan, polyacrylamide and GO, were successfully combined to form a stable porous composite. Additionally, the FT-IR results indicated that CAG had many functional groups, thus providing many adsorption sites for methylene blue. In addition, the TGA results showed that the decomposition temperature of CAG was 330.7 °C. This result indicated that CAG had high thermal stability.

(3) We have conducted a various of experiments by varying the GO proportion in CAG, initial MB concentration, adsorption temperature, adsorption time, quantity of CAGs and pH values of solutions. The results indicated that the GO proportion in CAG and the adsorption capacity were proportionally related, except when the GO proportion exceeded 25 wt% due to the instability of CAG. Additionally, the quantity of CAG and the removal rate exhibited a direct proportional relationship. While the number of active sites increased, they were inversely proportional to the adsorption capacity due to the decrease in the utilization rate.

(4) The adsorption isotherm of CAG for methylene blue (MB) was excellently depicted by the Langmuir model. After calculation, the theoretical maximum adsorption capacity should be 510.2 mg/g. An analysis of the kinetic model revealed that the adsorption was more fitted pseudo-second-order adsorption model.

(5) Adsorption thermodynamic analysis of CAG showed that Δ*H* ^0^ was negative. It’s revealed that adsorption process is an exothermic process, that is, the low temperature favored adsorption. The negative value of Δ*S* ^0^ proves that the adsorption of MB was a spontaneous adsorption process. Moreover, due to the value of Δ*S* ^0^ being negative, the adsorption of MB was a spontaneous adsorption process.

Therefore, the above conclusions show that CAG is an excellent adsorbent for organic dyes.

## Figures and Tables

**Table 1:** The specific quantity of each material in CAG synthesis.

Number	AM (g)	GO (mg)	CS (g)	KPS (mg)	TEMED (mL)	H_2_O (mL)	GO %
1	1	0	0.03	0.01	0.5	10	0%
2	1	54	0.03	0.01	0.5	10	5%
3	1	114	0.03	0.01	0.5	10	10%
4	1	184	0.03	0.01	0.5	10	15%
5	1	260	0.03	0.01	0.5	10	20%
6	1	325	0.03	0.01	0.5	10	25%

**Table 2:** Adsorption isotherm.

T/K	Langmuir				Freundlich		
-	*q_max_*	*k_L_*	*R* ^2^	*R_L_*	*k_F_*	1/*n*	*R* ^2^
-	(mg/g)	(L/mg)	-	-	(L/mg)	-	-
293 K	510.204	0.2253	0.9975	0.017–0.027	217.02	0.2064	0.9337
313 K	476.19	0.1871	0.9953	0.019–0.323	210.61	0.1815	0.8522
333 K	400.28	0.0564	0.9967	0.059–0.099	119.11	0.2289	0.9613

**Table 3:** The maximum adsorption capacity of MB by various materials.

Number	Sample	Maximum Adsorption Capacity	References
1	Activated lignin-chitosan	36.25 mg/g	[29]
2	polyacrylamide/GO hydrogel	255.48 mg/g	[30]
3	Graphene Oxide/Diatomite Composites	125 mg/g	[31]
4	Chlorine-functionalized reduced graphene oxide	221.4 mg/g	[32]
5	graphene oxide/calcium alginate composites	181.81 mg/g	[33]
6	polysaccharide-graphene oxide composite	358.4 mg/g	[34]
7	Graphene Oxide/Chitosan	468 mg/g	[35]
8	Graphene oxide/Fe_3_O_4_/chitosan nanocomposite	30.10 mg/g	[36]
9	Hydrolyzed Polyacrylamide Grafted Xanthan Gum and Incorporated Nanosilica	378.8 mg/g	[37]
10	Chitosan/Graphene Oxide Nanocomposites	662.25 mg/g	[38]

**Table 4:** Adsorption kinetics.

*C*_0_ (mg/L)	100	
Pseudo-first-order model	*k*_1_ (min^−1^)	0.023
	*q_e_* (mg/g)	258.22
	*R* ^2^	0.8868
Pseudo-second-order model	*k*_2_ (g/mg min)	4.65 × 10^−5^
	*q_e_* (mg/g)	500
	*R* ^2^	0.9998
Intraparticle diffusion model	*k_id_*_1_	52.539
	*c*	−54.51
	*R*_1_^2^	0.9952
	*k_id_*_2_	13.73
	*R*_2_^2^	0.9819
	*c*	202.7
	*k_id_*_3_	1.513
	*R*_3_^2^	0.7621
	*c*	410.43

**Table 5:** Adsorption thermodynamics.

*T*/K	Δ*G* ^0^ (kJ/mol)	Δ*H* ^0^ (kJ/mol)	Δ*S* ^0^ (J/mol)
293 K	−5.94	−33.2	−93.01
313 K	−4.05		
333 K	−2.23		
